# Factors influencing maternal-fetal attachment in pregnant women during the COVID-19 pandemic: a cross-sectional study

**DOI:** 10.4069/kjwhn.2023.02.21.3

**Published:** 2023-03-31

**Authors:** Hyeryeong Yoon, Hyunkyung Choi

**Affiliations:** 1 College of Nursing, Kyungpook National University, Daegu, Korea; 2 Research Institute of Nursing Science, Kyungpook National University, Daegu, Korea

**Keywords:** COVID-19, COVID-19 stress syndrome, Maternal-fetal relations, Pregnant women, Psychological resilience

## Abstract

**Purpose:**

Coronavirus disease 2019 (COVID-19) has spread widely throughout the world, causing psychological problems such as fear, anxiety, and stress. During the COVID-19 pandemic, pregnant women have been concerned about both their own health and the health of their fetuses, and these concerns could negatively affect maternal-fetal attachment. Thus, this study aimed to explore the level of COVID-19 stress, resilience, and maternal-fetal attachment among pregnant women during the COVID-19 pandemic, and to identify factors influencing maternal-fetal attachment.

**Methods:**

In total, 118 pregnant women past 20 weeks gestation were recruited from two maternity clinics in Daegu, Korea, to participate in this descriptive correlational study during COVID-19. The factors influencing maternal-fetal attachment were analyzed using hierarchical multiple regression analysis.

**Results:**

The mean scores for COVID-19 stress, resilience, and maternal-fetal attachment were 57.18±10.32 out of 84, 67.32±15.09 out of 100, and 77.23±9.00 out of 96, respectively. Nulliparous pregnant women reported greater maternal-fetal attachment than multiparous pregnant women (*p*=.003). Religious pregnant women also reported greater maternal-fetal attachment than non-religious pregnant women (*p*=.039). Resilience (β=.29, *p*=.002), COVID-19 stress (β=.20, *p*=.030) and parity (β=–.17, *p*=.047) were factors influencing maternal-fetal attachment, and these factors explained 26.4% of the variance in maternal-fetal attachment (F=10.12, *p*<.001).

**Conclusion:**

Converse to common sense, COVID-19 stress exerted a positive influence on maternal-fetal attachment in pregnant women during the COVID-19 pandemic. Healthcare providers need to recognize the positive influence of COVID-19 stress and implement intervention strategies to strengthen resilience in pregnant women to improve maternal-fetal attachment.

## Introduction

Coronavirus disease 2019 (COVID-19) has spread widely throughout the world since January 2020. As of October 2022, 615,301,980 cases and 6,524,568 deaths have been recorded worldwide [1]. In South Korea, 24,848,184 people have been infected, with women accounting for a higher percentage (53%) of reported cases than men (47%), and there have been 28,528 deaths [2]. COVID-19 causes various physical and mental symptoms and complications that can lead to psychological problems such as fear, anxiety, and posttraumatic stress disorder (PTSD) for confirmed cases, healthcare providers, and the general public [3,4].

According to an international study, the COVID-19 pandemic is a life stressor that contributes to psychological pain, and the resulting stress is a major risk factor for mental health problems in perinatal women [5]. Although the COVID-19 pandemic does not meet the diagnostic criteria for general PTSD, the emotional impact is similar to PTSD and can be traumatic. In particular, remembering these experiences can cause pregnant women to experience a higher level of PTSD symptoms and pain [6]. Pregnant women are concerned about the health of their fetus and their own health during the COVID-19 pandemic, which could negatively impact maternal-fetal attachment.

Maternal-fetal attachment is an indicator of the well-being of pregnant women and their fetuses. For the fetus, maternal-fetal attachment is both a physical bond and an emotional connection with the mother [7]. High maternal-fetal attachment has a positive influence on fetal brain development, autonomic nervous system development, children’s emotions and behaviors, and parent-child relationships [8,9]. For mothers, it is also an essential factor that affects the achievement of developmental tasks of pregnant women and the formation of successful mother-child relationships after childbirth [8]. However, an international study on Italian pregnant women reported that the high level of anxiety experienced during the COVID-19 pandemic negatively influenced the process of forming maternal-fetal attachment [10]. Therefore, it can be predicted that pregnant women may have difficulty forming maternal-fetal attachment during the COVID-19 pandemic, which could have negative impacts on the physical and mental health of both the mother and fetus.

Previous studies have reported a very diverse range of factors influencing maternal-fetal attachment, including religion and body image as general factors [11] and planned pregnancy and parity as obstetric factors [11,12]. Additionally, current health status, *taegyo* (traditional Korean prenatal care for fetus), sociability, and stress coping methods have been identified as relevant personal factors [9,13], and maternal-fetal attachment is also influenced by support system-related factors, such as social support, spouse support, and support from special others [12,14].

Among these influencing factors, stress has been found to have a negative impact on maternal-fetal attachment in most previous studies. Stress during pregnancy refers to unpleasant emotions such as anxiety, fear, and worry. A higher level of stress in pregnant women is associated with a lower quality of life [15], and stress during pregnancy lowers maternal role confidence [16] and negatively affects maternal-fetal attachment and the health of the fetus [9]. For example, depression and stress in pregnant women can cause low birth weight infants, preterm birth, stillbirth, and obstetric complications, and can permanently impede the growth and development of children [17]. In other words, stress during pregnancy can cause negative emotional changes, leading to lower maternal-fetal attachment [18].

As with pregnancy-related stress, stress due to COVID-19 is intense and comparable to posttraumatic stress [6]. Since pregnant women are vulnerable to COVID-19 due to reduced immunity in pregnancy [19], it can be assumed that pregnant women experience higher levels of COVID-19 stress than the general public. However, previous studies on COVID-19 stress in pregnant women are very limited. Since the impact of COVID-19 stress on pregnant women may be different from the impact of pregnancy stress, it is important to address this as a significant factor.

Stress adaptation can vary depending on how individuals cope with events, and resilience is one of the factors that positively influence methods of coping with stress [20]. Resilience refers to the quality or ability that enables individuals to adapt and recover on their own in serious situations, such as adversities and difficulties that may occur physically, mentally, emotionally, and socially in their lives [21]. In stressful situations, individuals with negative emotions tend to focus on the negative aspects rather than the positive aspects, leading to more stress. If negative emotions are not effectively resolved and are accumulated or repeated, they can cause anxiety and depression, which can lead to difficulties in forming maternal-fetal attachment [13]. Increasing self-management ability through discovering one’s strengths in stressful situations is one way to increase resilience [22]. Therefore, it can be assumed that resolving negative emotions through resilience and seeking better adaptation methods may increase maternal-fetal attachment. Pregnant women strive to reduce stress and anxiety during pregnancy to maintain the health of the fetus, complement negative emotions with positive emotions, and avoid events that may cause problems, and these efforts strengthen resilience [23]. Based on the results of previous studies, it can be inferred that resilience helps reduce stress and positively influences maternal-fetal attachment.

The COVID-19 situation is changing rapidly, and the stress of pregnant women is accumulating due to the prolonged infectious disease pandemic. Although previous studies have extensively investigated the factors influencing maternal-fetal attachment, domestic and international studies on maternal-fetal attachment in stressful situations such as COVID-19 are very limited. Therefore, it is necessary to investigate the influence of COVID-19 stress and resilience on maternal-fetal attachment. This study aimed to identify the influence of COVID-19 stress and resilience on maternal-fetal attachment in a special situation, the COVID-19 pandemic, and to provide basic data for developing measures and intervention strategies to improve maternal-fetal attachment.

The purpose of this study was to determine the level of COVID-19 stress, resilience, and maternal-fetal attachment of prenatal pregnant women during the COVID-19 pandemic and to identify the influence of those factors on maternal-fetal attachment. The detailed objectives of the study were as follows:

1) To determine the level of maternal-fetal attachment according to the general and obstetric characteristics of pregnant women

2) To determine the levels of COVID-19 stress, resilience, and maternal-fetal attachment and to examine the relationships among those variables

3) To identify the factors that influenced maternal-fetal attachment in pregnant women

## Methods

Ethics statement: This study was approved by the Institutional Review Board of Kyungpook National University (2022-0215). Informed consent was obtained from the participants.

### Study design

This descriptive correlational study was conducted to determine the levels of COVID-19 stress, resilience, and maternal-fetal attachment among pregnant women during the COVID-19 pandemic and to identify the factors that influenced maternal-fetal attachment. This study adhered to the STROBE reporting guidelines (http://www.strobe-statement.org).

### Sample and sampling

Participants were recruited by convenience sampling from two women’s clinics located in Daegu, Korea. The selection criteria were pregnant women at 20 or more weeks of gestation who could feel fetal movements, understood the study’s purpose, and consented to participate in the study voluntarily, and who could understand and respond to the questionnaire. The exclusion criteria were pregnant women diagnosed with high-risk pregnancy, had a fetus with congenital deformities or severe complications, diagnosed with depression during data collection, taking antidepressants or anxiolytics, international marriage migrants, or non-Koreans. The number of participants was calculated using G*Power 3.1.9.7. With a significance level of .05, a power of .80, median effect size of .15, and 10 predictors (COVID-19 stress, resilience, and eight general and obstetric characteristics excluding COVID-19-related characteristics), at least 118 participants were required. Considering a dropout rate of 10%, 130 participants were recruited through convenience sampling. Excluding 12 questionnaires with incomplete responses, 118 questionnaires were used for data analysis.

### Measurements

All the measurements used in this study were approved by the measurement developers and translators into Korean.

#### COVID-19 stress

This study utilized the COVID-19 Stress Scale for Korean People (CSSK) developed by Kim et al. [24]. The tool consists of 21 items that are divided into three factors: fear of infection, difficulties of social distancing, and anger toward others. Each item is scored using a 5-point Likert scale (0–4 points). The score range is 0–84 points, and a higher total score indicates a higher level of COVID-19 stress. Cronbach’s α was .96 at the time of development and .86 in this study.

#### Resilience

Resilience was measured using the Korean version of the Connor Davidson Resilience Scale (CD-RISC), which was originally developed by Connor and Davidson [21]. The CD-RISC consists of 25 items, and each item is scored using a 5-point Likert scale (0–4 points). The score range is 0–100 points, and a higher total score indicates higher resilience. Cronbach’s α was .89 at development and .96 in this study.

#### Maternal-fetal attachment

The Maternal-Fetal Attachment Scale (MFAS), originally developed by Cranley [8] and translated into Korean and modified by Kim [25], was also used in this study. The MFAS consists of 24 items, and each item is scored using a 4-point Likert scale (1–4 points). The score range is 24–96 points, and a higher total score indicates stronger maternal-fetal attachment. Cronbach’s α was .85 upon development, .89 in Kim’s study [25], and .88 in this study.

#### General and obstetric characteristics

The general and obstetric characteristics were developed by the researchers based on the literature [11,26] and included age, religion, educational level, occupation, COVID-19 experience of pregnant women, COVID-19 experience in the family, gestational period, parity, planned pregnancy, and pregnancy method.

### Data collection procedures

Data were collected in July 2022, which was when the Omicron variant resurgence was observed. The chiefs of nursing departments at two women’s clinics in Daegu were briefed on the study’s purpose and methods, and cooperation was requested for data collection. The survey was conducted while the participants were waiting for medical treatment. After checking the selection and exclusion criteria, only those who voluntarily agreed to participate in the study and provided written consent were included in the data collection. Participants were provided with an explanation of the study’s purpose, participation period, potential side effects, risk factors, safety measures, benefits and compensation, personal information protection, and confidentiality. Also, we emphasized that participation was voluntary and the right to withdraw from the study at any time. The researchers also provided their contact information in case participants had any questions. In consideration of the COVID-19 pandemic, the participants and researchers wore KF-94 masks, and the researcher explained the study details face-to-face while maintaining a distance of at least 1 meter. Participants used their own pens, and new pens were provided if needed. After completing the questionnaire, participants placed it in an individual envelope and handed it to the researcher. The questionnaire took approximately 10 minutes to complete, and a small gift (worth Korean won 5,000; approximately 3.81 USD) was given to participants.

### Data analysis

The collected data were analyzed using SPSS for Windows ver. 24.0 (IBM Corp., Armonk, NY, USA). The general and obstetric characteristics of pregnant women were presented as frequency, percentage, mean, and standard deviation. The degree of COVID-19 stress, resilience, and maternal-fetal attachment of pregnant women was presented using the mean and standard deviation. The *t*-test and one-way analysis of variance were used to analyze differences in maternal-fetal attachment according to the general and obstetric characteristics of pregnant women, and *post hoc* analysis was conducted using the Scheffé test. Pearson correlation coefficients were used to analyze the correlations between COVID-19 stress, resilience, and maternal-fetal attachment. Finally, hierarchical multiple regression analysis was conducted to analyze the factors influencing maternal-fetal attachment in pregnant women.

## Results

### General and obstetric characteristics of pregnant women

The majority of the pregnant women (n=71, 60.2%) were between the ages of 30 and 34 years. Seventy-three (61.9%) pregnant women were not religious, 78 (66.1%) had graduated from university or higher, and 52 (44.1%) were actively employed. Nearly half of the pregnant women (n=58, 49.2%) had experienced a COVID-19 infection, and 73 (61.9%) had family members who had experienced a COVID-19 infection. The most common gestational period was between 26 and 30 weeks (n=36, 30.5%). Most of the pregnant women (n=77, 65.3%) were nulliparous and 79 (66.9%) had planned their pregnancy. Most participants (n=102, 86.4%) had conceived spontaneously (Table 1).

### Levels of COVID-19 stress, resilience, and maternal-fetal attachment

A high level of COVID-19 stress (57.18±10.32 points), a moderate level of resilience (67.32±15.09 points), and a high level of maternal-fetal attachment (77.23±9.00 points) were confirmed among the pregnant women (Table 2).

### Differences in maternal-fetal attachment according to the general and obstetric characteristics of pregnant women

In the analysis of differences in maternal-fetal attachment scores according to the general characteristics of pregnant women, religion (t=2.09, *p*=.039) showed a statistically significant difference. More specifically, the score of maternal-fetal attachment was higher among pregnant women with religion than those without religion. Among obstetric characteristics, parity (t=3.06, *p*=.003) demonstrated a statistically significant difference, and nulliparous pregnant women showed higher scores of maternal-fetal attachment than multiparous pregnant women (Table 1).

### Correlations among COVID-19 stress, resilience, and maternal-fetal attachment in pregnant women

Maternal-fetal attachment in pregnant women was found to have a weak positive correlation with COVID-19 stress (r=.37, *p*<.001) and a moderate positive correlation with resilience (r=.44, *p*<.001). These findings indicated that higher levels of COVID-19 stress and resilience were associated with higher maternal-fetal attachment (Table 3).

### Factors influencing maternal-fetal attachment in pregnant women

To identify the factors influencing maternal-fetal attachment in pregnant women, two-step hierarchical regression analysis was conducted. In the first step, religion and parity, which were general and obstetric characteristics that showed statistically significant relevance with maternal-fetal attachment in the univariate analysis, were entered into the model. In the second step, the net effects of COVID-19 stress and resilience on maternal-fetal attachment were analyzed by inputting COVID-19 stress and resilience while controlling for general and obstetric characteristics. The Durbin-Watson index was 2.02, indicating no autocorrelation among error terms, and the variance inflation factor was 1.06–1.35, confirming no multicollinearity between independent variables. Finally, the normality of independent variables was confirmed through a normal probability plot of the residuals. In the first step, parity (β=–.25, *p*=.006) was a statistically significant influencing factor and explained 9.9% of maternal-fetal attachment. In the second step, resilience (β=.29, *p*=.002), COVID-19 stress (β=.20, *p*=.030), and parity (β=–.17, *p*=.047) were found to be factors influencing maternal-fetal attachment in pregnant women, with an explanatory power of 26.4% (F=10.12, *p*<.001) (Table 4).

## Discussion

The mean score for COVID-19 stress in this study was 78.18 out of 105 points, which was similar to the score of 72.87 points reported in a previous study on the effects of mothers’ COVID-19 stress on young children’s problem behaviors using the same tool [27]. Although a direct comparison is difficult due to differences in measurement tools, a national mental health survey conducted by the Korean Society for Traumatic Stress Studies in June 2022 reported a score of 11.16 out of 27 points for “fear of infection,” a subcategory of the CSSK, indicating that the COVID-19 stress of pregnant women in this study was much higher than in the general population [28]. Additionally, a previous study on the stress levels of Polish pregnant women during the COVID-19 pandemic, which used the Perceived Stress Scale, found a score of 18 out of 40; this score was lower than 50% of the maximum possible score and indicated a moderate level of stress [29]. These results suggest that COVID-19 stress in Korean pregnant women is higher than that in pregnant women from other countries.

Although caution is necessary when making comparisons due to insufficient studies on COVID-19 stress of Korean pregnant women during the pandemic, this study’s results can be interpreted based on previous research on women in their 20s to 40s due to the age range of participants. Firstly, a previous study on COVID-19 stress among university students reported a score of 78.00 out of 105 points [30], which was similar to the score in the current study. Secondly, a study on the effect of COVID-19 stress on psychological burnout of special education teachers found a mean score of 3.62 points [31], equivalent to 76.02 points on a 105-point scale, which was comparable to the current study’s findings. Female university students and female teachers have been shown to experience higher COVID-19 stress than their male counterparts, respectively [30,31], suggesting that women are more susceptible to COVID-19 stress than men. Additionally, an international study on posttraumatic growth of pregnant women during the COVID-19 pandemic found that socially marginalized and vulnerable groups, such as pregnant women, experienced higher levels of stress and psychological pain [32]. Specifically, adults aged 35 years or younger and women experienced more stress during the pandemic [33], and women experienced more negative emotions, such as anxiety and fear, during this period [34]. Although it is challenging to make direct comparisons due to the lack of domestic studies on COVID-19 stress of pregnant women using the same tool as this study, these findings indicate the need for increased attention to COVID-19 stress among pregnant women and women who may become pregnant.

Religion was found to be the only significant factor among the general characteristics of pregnant women, with religious pregnant women exhibiting significantly higher maternal-fetal attachment. This finding is consistent with previous studies on factors influencing maternal-fetal attachment in pregnant women [11] and women who had a miscarriage [35]. It is suggested that religious activities help pregnant women to offset negative emotions and strengthen their positive relationship with the fetus. Religious beliefs emphasizing the preciousness of life may also favorably impact maternal-fetal attachment. Therefore, even if face-to-face activities are difficult, encouraging non-face-to-face religious activities such as real-time broadcasting to help generate positive emotions in pregnant women may improve maternal-fetal attachment.

Among the obstetric characteristics, parity was significantly associated with maternal-fetal attachment, with nulliparous pregnant women exhibiting higher maternal-fetal attachment than multiparous pregnant women. This finding is inconsistent with a previous study on pregnant women of advanced maternal age, which found that those who had experienced two or more childbirths exhibited higher maternal-fetal attachment than those who had not given birth or had experienced only one childbirth [12]. However, the current study’s results are consistent with a previous study on the relationship between prenatal stress and maternal-fetal attachment, according to which nulliparous pregnant women exhibited higher maternal-fetal attachment than multiparous women [18]. This may be due to the fact that nulliparous pregnant women are having their first children, and they are sensitively caring for the fetuses with particular affection and attention. Despite these results, the differences from previous studies suggest the need for replication studies on the relationship between childbirth experience and maternal-fetal attachment.

Resilience was found to have a positive correlation with maternal-fetal attachment in this study, and it was also identified as a major factor influencing maternal-fetal attachment. The relationship between these two variables can be explained by the fact that a series of positive emotions during pregnancy promote emotional growth in the brain, increase serotonin levels, and release pleasure hormones for the mother and ultimately in the fetus, leading to decreased maternal anxiety and the formation of stable maternal-fetal attachment [23]. The resilience questionnaire used in this study included questions about adaptability, confidence in dealing with challenges and adversities, and self-esteem, which are key components of resilience. This finding is consistent with previous studies that have shown that higher self-esteem of pregnant women was associated with higher maternal-fetal attachment [11], and that higher parental efficacy was associated with higher maternal-fetal attachment [36]. Since resilience can be improved through self-management in stressful situations by discovering one's individual strengths [22], it is considered that maternal-fetal attachment is enhanced through the process of self-control and adaptation to a new role as a mother despite external adversities.

Converse to common sense, this study also found that COVID-19 stress in pregnant women was a positive factor influencing maternal-fetal attachment, and higher COVID-19 stress was associated with greater maternal-fetal attachment. This result is similar to studies on other types of stress during pregnancy, such as a previous study that found a positive correlation between preterm labor stress and maternal-fetal attachment [37], and an international study that found an increase in maternal-fetal attachment as pregnant women perceived more psychosocial stress [38]. Although a direct comparison is difficult due to a lack of studies on the correlation between maternal-fetal attachment and COVID-19 stress, the results of this study indicate that COVID-19 stress does not always have a negative effect on the relationship between a mother and her fetus. For instance, an international study found that maternal-fetal attachment in high-risk pregnant women was higher than normal pregnant women, and high-risk situations such as high-risk pregnancies increased adaptation to pregnancy and strengthened maternal-fetal attachment [39]. Furthermore, previous studies have suggested that COVID-19 stress could lead to an increase in maternal-fetal attachment by wanting to care more for the fetus and provide protection [40]. Childbirth during traumatic situations like the COVID-19 pandemic has also been reported to trigger maternal psychological growth by presenting feelings of gratitude for life and individual capacity, and thus bringing about better mother-infant attachment [41]. Based on these findings, it can be inferred that even during the COVID-19 pandemic, pregnant women experience changes from womanhood to motherhood, feel gratitude for life and their ability to overcome adversity through the fetus, rather than perceiving COVID-19 stress negatively; thus, transforming COVID-19 stress into a positive concept toward maternal-fetal attachment. However, as other studies [15,16] reported pregnancy stress negatively influencing maternal-fetal attachment, the possibility that COVID-19 stress could negatively impact maternal-fetal attachment also cannot be ruled out, and stress should be monitored. Future research should investigate this issue. Furthermore, a previous study has already investigated COVID-19 stress and pregnancy stress separately and emphasized the importance of reducing both types of stress [42]. Considering the reports of previous studies that pregnant women experienced increased maternal-fetal attachment over time [43] and it was not negatively affected by the COVID-19 pandemic [44], it seems necessary to consider COVID-19 stress separately from pregnancy stress.

Since this study was conducted in a limited number of maternity clinics in a particular region, generalization of the results should be approached with caution. Additionally, this study was conducted during the resurgence of the Omicron variant, and the first death of a pregnant woman confirmed with COVID-19 in January 2022 [45] may have increased the weight of COVID-19 stress on pregnant women. The Omicron variant was first reported in South Korea in December 2021 [46], and the Korea Disease Control and Prevention Agency announced the spread of the Delta variant in August 2021 [47]. Social countermeasure recommendations were changing depending on the type of variant, and South Korea was preparing to release regulations regarding wearing masks indoors [48]. Therefore, since the level of COVID-19 stress in pregnant women can differ depending on the effects of case fatality rate and transmission of the variant, and social coping, it is important to interpret the results of this study carefully. Studies investigating the relationships between COVID-19 stress, resilience, and maternal-fetal attachment of pregnant women in the context of the COVID-19 pandemic are insufficient, and more studies are necessary to confirm those relationships.

In conclusion, while many studies have investigated variables related to maternal-fetal attachment, this study is significant in that it investigated COVID-19 stress and resilience in pregnant women in relation to maternal-fetal attachment, which has rarely been explored in previous studies. Moreover, this study confirmed that strategies to strengthen resilience are necessary when developing programs to enhance maternal-fetal attachment during infectious disease situations similar to COVID-19, and it presents a new perspective according to which COVID-19 stress can be positively sublimated, rather than being viewed only negatively. Healthcare providers can use these findings to assess stress related to COVID-19 and/or future widespread infectious conditions, while considering its positive influence on maternal-fetal attachment. Further COVID-19 studies that include pregnant women from other domestic and international regions, high-risk pregnant women, and long-term hospitalized pregnant women are needed. Also, practical educational programs that strengthen resilience to enhance maternal-fetal attachment are needed for future widespread infectious conditions.

## Figures and Tables

**Table 1.** Differences in maternal-fetal attachment according to general and obstetric characteristics (N=118)

Characteristic	Categories	n (%)	Maternal-fetal attachment	
			Mean±SD	t/F (*p*)
Age (year)	≤30	26 (22.0)	77.81±9.81	0.46 (.630)
	31–34	71 (60.2)	77.52±9.12	
	≥35	21 (17.8)	75.52±7.61	
Religion	Yes	45 (38.1)	79.40±8.55	2.09 (.039)
	No	73 (61.9)	75.89±9.06	
Level of education	≤College	40 (33.9)	75.43±9.18	–1.57 (.119)
	≥Bachelor’s	78 (66.1)	78.15±8.82	
Occupation	Actively employed	52 (44.1)	78.40±8.44	0.87 (.420)
	Parental leave	42 (35.6)	76.64±9.80	
	Not employed	24 (20.3)	75.71±8.74	
COVID-19 experience	Yes	58 (49.2)	76.69±10.73	–0.63 (.572)
	No	60 (50.8)	77.75±6.97	
COVID-19 experience in family	Yes	73 (61.9)	77.45±9.55	0.34 (.733)
	No	45 (38.1)	76.87±8.12	
Gestational period (week)	20–25	27 (22.9)	76.45±9.23	0.20 (.897)
	26–30	36 (30.5)	76.72±9.37	
	31–35	27 (22.9)	78.15±8.10	
	36–40	28 (23.7)	77.64±9.71	
Parity	0	77 (65.3)	79.01±8.02	3.06 (.003)
	≥1	41 (34.7)	73.88±9.84	
Planned pregnancy	Yes	79 (66.9)	77.44±9.26	0.37 (.714)
	No	39 (33.1)	76.79±8.55	
Pregnancy method	Spontaneous	102 (86.4)	77.00±9.44	–1.04 (.306)
	Ovulation induction	16 (13.6)	78.69±5.30	

COVID-19: Coronavirus disease 2019.

**Table 2.** Levels of COVID-19 stress, resilience, and maternal-fetal attachment (N=118)

Variable	Range	Min	Max	Mean±SD
COVID-19 stress	0–84	32	75	57.18±10.32
Resilience	0–100	38	95	67.32±15.09
Maternal-fetal attachment	24–96	53	93	77.23±9.00

COVID-19: Coronavirus disease 2019.

**Table 3.** Correlations among COVID-19 stress, resilience, and maternal-fetal attachment (N=118)

Variable	R (*p*)	
	COVID-19 stress	Resilience
COVID-19 stress	1	
Resilience	.47 (<.001)	1
Maternal-fetal attachment	.37 (<.001)	.44 (<.001)

COVID-19: Coronavirus disease 2019.

**Table 4.** Factors influencing maternal-fetal attachment (N=118)

Step	Categories	B	SE	β	t (*p*)	R^2^ (ΔR^2^)	F (*p*)
1	(Constant)	88.30	3.36		26.32 (<.001)	.10 (.099)	6.29 (.003)
	Religion^†^	–2.89	1.65	–.16	–1.76 (.082)		
	Parity^†^	–4.74	1.68	–.25	–2.82 (.006)		
2	(Constant)	61.85	6.15		10.06 (<.001)	.26 (.165)	10.12 (<.001)
	Religion^†^	–1.35	1.53	–.07	–0.88 (.382)		
	Parity^†^	–3.14	1.57	–.17	–2.00 (.047)		
	COVID-19 stress	0.18	0.08	.20	2.20 (.030)		
	Resilience	0.18	0.06	.29	3.12 (.002)		

COVID-19: Coronavirus disease 2019.

† The reference groups were religion (yes) and parity (0).
